# Diagnosis of *PTEN* mosaicism: the relevance of additional tumor DNA sequencing. A case report and review of the literature

**DOI:** 10.1186/s12920-023-01600-0

**Published:** 2023-07-13

**Authors:** Mathias Cavaillé, Delphine Crampon, Viorel Achim, Virginie Bubien, Nancy Uhrhammer, Maud Privat, Flora Ponelle-Chachuat, Mathilde Gay-Bellile, Mathis Lepage, Zangbéwendé Guy Ouedraogo, Natalie Jones, Yannick Bidet, Nicolas Sevenet, Yves-Jean Bignon

**Affiliations:** 1 Imagerie Moléculaire Et StratégiesThéranostiques, Université Clermont Auvergne, INSERM, U1240, 63000 Clermont Ferrand, France; 2 Département d’Oncogénétique, Centre Jean Perrin, 63011 Clermont-Ferrand, France; 3 Service d’hépato-Gastro-Entérologie, Pôle Santé République, 63000 Clermont Ferrand, France; 4 Service de Neurochirurgie, CHU Gabriel Montpied, 63000 Clermont Ferrand, France; 5 Unité d’oncogénétique, Institut Bergonié, 229 Cours de L’Argonne, 33076 Bordeaux Cedex, France; 6 INSERM U1218, Institut Bergonié, 229 Cours de L’Argonne, 33076 Bordeaux Cedex, France; 7 UFR de Pharmacie, Univ. Bordeaux, 146 Rue Léo Saignat, 33076 Bordeaux Cedex, France

**Keywords:** *PTEN*, Cowden syndrome, Mosaicism, Tumoral sequencing, NGS sequencing

## Abstract

**Background:**

PTEN hamartoma syndrome (PHTS) is an autosomal dominant disorder characterized by pathogenic variants in the tumor suppressor gene phosphatase and tensin homolog (*PTEN*). It is associated with an increased risk of muco-cutaneous features, hamartomatous tumors, and cancers. Mosaicism has been found in a few cases of patients with de novo PHTS, identified from blood samples. We report a PHTS patient with no variant identified from blood sample. Constitutional PTEN mosaicism was detected through sequencing of DNA from different tumoral and non-tumoral samples.

**Case presentation:**

Our patient presented clinical Cowden syndrome at 56 years of age, with three major criteria (macrocephaly, Lhermitte Duclos disease, oral papillomatosis), and two minor criteria (structural thyroid lesions, esophageal glycogenic acanthosis). Deep sequencing of *PTEN* of blood leukocytes did not reveal any pathogenic variants. Exploration of tumoral (colonic ganglioneuroma, esophageal papilloma, diapneusia fibroids) and non-tumoral stomach tissues found the same *PTEN* pathogenic variant (NM_000314.4 c.389G > A; p.(Arg130Gln)), with an allelic frequency of 12 to 59%, confirming genomic mosaicism for Cowden syndrome.

**Conclusions:**

This case report, and review of the literature, suggests that systematic tumor analysis is essential for patients presenting *PTEN* hamartoma syndrome in the absence of any causal variant identified in blood leukocytes, despite deep sequencing. In 65 to 70% of cases of clinical Cowden syndrome, no pathogenic variant in the *PTEN* is observed in blood samples: mosaicism may explain a significant number of these patients. Tumor analysis would improve our knowledge of the frequency of de novo variations in this syndrome. Finally, patients with mosaicism for *PTEN* may not have a mild phenotype; medical care identical to that of heterozygous carriers should be offered.

## Background

PTEN hamartoma syndrome (PHTS) is an autosomal dominant disorder characterized by pathogenic variants in the tumor suppressor gene phosphatase and tensin homolog (*PTEN*) [1]. The clinical presentation is heterogeneous, including Cowden syndrome (CS) [2], Bannayan-Riley-Ruvalcaba syndrome (BRRS) [3], Lhermitte–Duclos disease [4], Segmental outgrowth-lipomatosis-arteriovenous malformation-epidermal nevus (SOLAMEN) syndrome [5], and autism-macrocephaly syndrome (ASD) [6].

The prevalence of CS, probably underestimated, is estimated at 1 in 200,000 individuals [7], with a penetrance of up to 90% in the second decade [8]. It is associated with an increased risk of muco-cutaneous features, hamartomatous tumors, and cancers (Table 1) [9–11].

**Table 1:** Revised *PTEN* hamartoma tumor syndrome (PHTS) diagnostic criteria

Revised *PTEN* Hamartoma Tumor Syndrome (PHTS) Clinical Diagnostic Criteria
**Operational Diagnosis in an Individual (either of the following):**
(1) Three or more major criteria, but one must include macrocephaly, Lhermitte–Duclos disease, or gastrointestinal hamartomas; OR
(2) Two major and three minor criteria;
**Operational Diagnosis in a Family where One Individual Meets Revised PHTS Clinical Diagnostic Criteria or has a *PTEN* Mutation:**
(1) Any two major criteria with or without minor criteria; OR
(2) One major and two minor criteria; OR
(3) Three minor criteria
**Major Criteria**
Breast cancer
Endometrial cancer (epithelial)
Thyroid cancer (follicular)
Gastrointestinal hamartomas (including ganglioneuromas, but excluding hyperplastic polyps) (≥ 3)
Lhermitte–Duclos disease (LDD), adult
Macrocephaly (≥ 97 percentile)
Macular pigmentation of the glans penis
Multiple mucocutaneous lesions (any of the following):
Multiple trichilemmomas (≥ 3), at least one proven biopsy
Acral keratoses (≥ 3 palmoplantar keratotic pits and/or acral hyperkeratotic papules)
Mucocutaneous neuromas (≥ 3)
Oral papillomas (particularly on tongue and gingiva), multiple (≥ 3) OR biopsy-proven OR dermatologist-diagnosed
**Minor Criteria**
Autism spectrum disorder
Colon cancer
Esophageal glycogenic acanthosis (≥ 3)
Lipomas (≥ 3)
Mental retardation (i.e., Intelligence Quotient (IQ) ≤ 75)
Renal cell carcinoma
Testicular lipomatosis
Thyroid cancer (papillary or follicular variant of papillary)
Thyroid structural lesions (e.g., adenoma, multinodular goiter)
Vascular anomalies (including multiple intracranial developmental venous anomalies)

[12]

Mosaicism has been found in a few cases of patients with de novo PHTS [13]. This mosaicism can sometimes be identified in blood samples, using different techniques including deep next generation sequencing (NGS).

We present a patient with clinical CS, with no identified pathogenic variation in *PTEN* through deep sequencing of DNA from blood samples. Constitutional *PTEN* mosaicism was detected through sequencing of DNA from different tumoral and non-tumoral tissues. We review PHTS mosaicism reported in the literature, and suggest that mosaicism be actively searched for when possible.

## Case presentation

A 56-year-old man presented left-sided hyperacusis and loss of ipsilateral stapedial reflex. A cerebral MRI was performed and identified an infiltrative mass 57 mm in diameter located in the left cerebellar hemisphere, without enhancement of the contrast product, suggestive of a hamartomatous lesion of the posterior fossa.

Given the absence of symptoms, in particular of intracranial hypertension on ophthalmological examination, a control MRI was performed 3 months later. This second MRI found a characteristic appearance of Lhermitte Duclos disease (dysplastic gangliocytoma) of the left cerebellar hemisphere, with a striated lamellar appearance, measuring 60 mm × 36 mm × 31 mm. This lesion was associated with a mass effect on the lower part of the fourth ventricle, resulting in moderate triventricular hydrocephalus (Evans Index at 0.35), with slight signs of transpedicular cerebrospinal fluid (CSF) resorption.

The patient’s medical records did not report any delay in psychomotor development or learning difficulties. He had a history of “diapneusia fibroids” of the tongue that had been treated surgically some years earlier, and high blood pressure which was well-controlled by a combination of Angiotensin II antagonist and calcium channel blocker. The clinical examination found macrocephaly, with a head circumference of 60 cm (+ 2SD). Cutaneous-mucosal examination identified seborrheic keratosis in the shoulders and in the extremities. It also found fibrous lesions in interdental positions 31–32 and 32–33, as well as on the right lateral edge of the tongue. No cutaneous trichilemmomas or papules were found.

In this context, a check-up for other lesions was performed. Thyroid ultrasound found multinodular hypertrophy of the right lobe with five nodular and cystic lesions (3 to 15 mm in diameter), Eu-thirads 3. Esogastroduodenal fibroscopy found oesophageal papillomatosis, confirmed histologically, and hyperplastic polyps of the gastric antrum. Colonoscopy identified a 6-mm-diameter ganglioneuroma of the transverse colon, confirmed histologically.

The patient’s family history was not suggestive of PHTS, with no notable history. A clinical diagnosis of CS was made, due to the presence of three major criteria: Lhermitte Duclos disease in adult, macrocephaly, and oral papillomatosis. Two minor criteria were also present, including thyroid structural lesions and esophageal glycogenic acanthosis, as well as a unique digestive ganglioneruoma.

## Methods

To confirm the clinical diagnosis, constitutional and tumor analyses of the *PTEN* gene (NM_000314.4) were performed.

### Samples

DNA was extracted from peripheral blood using the QIAamp DNA Blood maxikit (Qiagen) and from oesophageal papilloma, ganglioneuroma, diapneusia fibroid and non-tumoral stomach tissue formalin-fixed paraffin-embedded blocks, using the Maxwell 16 FFPE Plus LEV kit (Promega). Libraries were prepared using KAPA HyperPlus Kits and captured using KAPA HyperCap Target Enrichment (Roche). The quality of libraries and captures was controlled with a Tapestation 4150 (Agilent). Sequencing was performed with a NextSeq 500/550 High Output Kit v2.5 (300 Cycles) on a NextSeq 550 instrument (Illumina). The percentage of tumor cells from the colon sample was 60%. The tumor surface of the esophageal sample, diapneusia fibroid and non-tumoral stomach tissue was respectively 16 mm^2^, 6 mm^2^ and 2 mm^2^.

### Bio-informatic analysis

De-multiplexing was performed using bcl2fastq2 Conversion Software (Illumina). Alignment was performed on University of California Santa Cruz human genome reference build 19 using the Burrows-Wheeler Aligner (v0.7.17). Genome Analysis Toolkit (GATK v 4.1.8.0) and PICARD (v 2.22.8) tools were used for base quality score recalibration (BaseRecalibrator) and identified duplicate reads (MarkDuplicates). Variant calling was performed by GATK HaplotypeCaller and by GATK Mutect and Freebayes (v1.2.0). Annotations were added by Ensembl VariantEffectPredictor (v96.0) and Annovar (8 Jun 2020). Copy number variation analysis was performed using ExomeDepth (v 1.1.12).

The entire coding sequence and intron/exon junctions (positions up to -20 and + 6) of *PTEN* gene were analyzed in all samples (blood, tumoral and non-tumoral). The entire 5'UTR and 3'UTR regions up to position *20 were analyzed from blood samples only. Due to the clinical presentation, no other gene was analyzed from all samples explored. Variants were filtered on mapping quality, call quality and minimum depth of coverage of 30 × for blood sample and 200 × for tumor samples. The detection threshold for constitutional analysis was 20% of reads. The detection threshold for tumor analyses was 10% of reads. The technical sensitivity was greater than 95%.

Variant interpretation was performed using ALAMUT Visual 2.11 (Interactive BioSoftware), and relevant databases (ClinVar). Splice variants were interpreted using SpliceAI (v1.3) and SPIP (v1.0) softwares. Variants were classified according to the American College of Medical Genetics (ACMG) recommendations.

## Results

NGS sequencing from two independent blood samples was first performed. The average depth for *PTEN* was 1633 with 100% coverage at 30X, 98.5% at 100X and 77.61% at 1000X. No constitutional variation of the *PTEN* gene was found from blood samples.

**Fig. 1:**
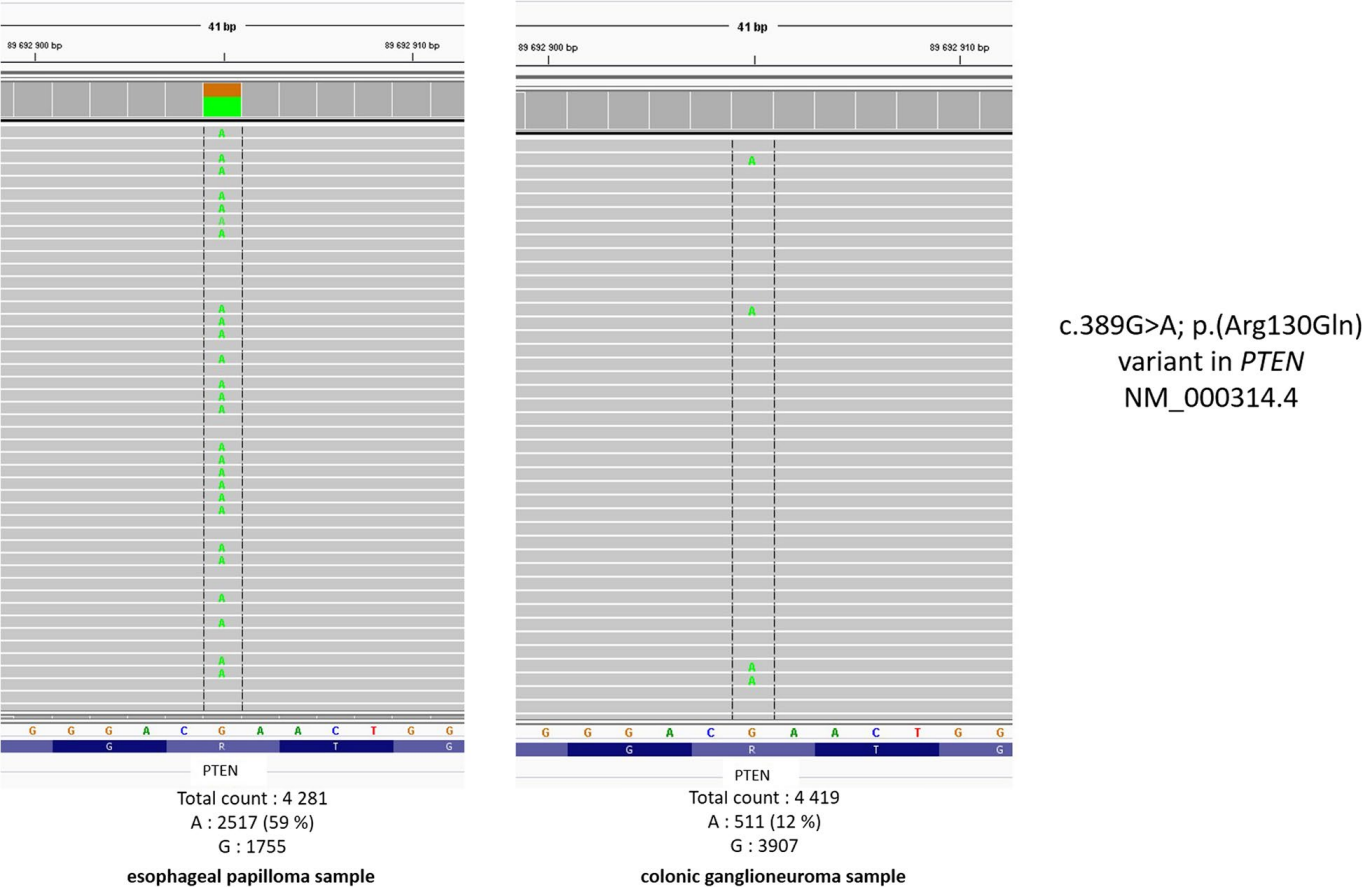
Variant c.389G > A; p.(Arg130Gln) found in mosaic in two tumoral sample in *PTEN* gene (NM_000314.4)

Given the strong clinical suspicion, and the absence of a probable pathogenic or pathogenic variant identified in *PTEN* from the blood samples, two tumor samples were analyzed (oesophageal papilloma, ganglioneuroma). The average depth was 3807 with 100% coverage. at 1000X and 95% at 1500X. The same pathogenic variant, c.389G > A; p.(Arg130Gln), was identified in both samples. The variant allele frequency was 12% in the ganglioneuroma sample (tumor cell content 60%) and 59% in the esophageal papilloma sample (tumor cell content unknown) (Fig. 1). To confirm the presence of constitutional mosaic *PTEN* in the patient and rule out the hypothesis that the same mutation occurred in two different tissues in an acquired process, two supplemental samples were expored, including one non-tumoral sample (diapneusia fibroid and non-tumoral stomach tissue). The same pathogenic variant was found with an allele frequency respectively at 25% and 14%, confirming the diagnosis.

This variant is located in the phosphatase functional domain of the protein. It is a recurrent pathogenic variation, involved in different clinical presentations associated with *PTEN* hamartoma tumor syndrome.

In addition, an acquired unknown significant variant c.816C > G, p.(His272Gln) was found in ganglioneuroma and papilloma tumoral samples, with allelic frequency respectively at 11 and 8%.

## Discussion and conclusions

Mosaicism is very rare in PTHS and only a few cases have been published. Most of these were reported in the study of Rofes et al.[13]*,* that included six patients*,* and three another cases were reported in the studies of Steffan et al. and Hendricks et al. [13–20] (Table 2).

**Table 2:** Mosaic *PTEN* patients reported in literature

Publications	Phenotype	Diagnosis Criteria	PTEN	PTEN variant	Genetic analysis		
					Technique	Blood Allele frequency	Somatic Allele Frequency
Gammon et al. (2013) [15]	CS	yes	NM_000314.6	c.966A > G; 967delA	Sanger	< 10%	NA
Pritchard et al. (2013) [16]	CS	yes	NM_000314.6	c.767_768delAG; p.(Glu256Val fs*41)	NGS (depth > 1000X)	1.70%	25–50% (skin, cerebellar, colonic, endocervical tissues) using Sanger
Salo-Mullen et al. (2013) [17]	CS	yes	NI	PTEN partial deletion (at least exons 6–9)	aCGH, MLPA	47 ± 10% of cells	NA
Steffan et al. (2014) [14]	CS	yes	NM_000314.6	c.675 T > A; p.(Tyr225*)	Sanger	< 20%	NA
Golas et al. (2018) [18]	BRRS	yes	NM_000314.6	10q23.1q23.3 deletion	aCGH, MLPA	50%	buccal mucosa
Golas et al. (2018) [18]	BRRS	yes	NM_000314.6	10q23.1q23.3 deletion	Sanger, aCGH, MLPA	< 10%	thyroid, colonic polyp
Goldenberg et al. (2019) [19]	ASD	yes	NM_000314.6	c970dup; p.(Asp324Gly fs*3)	NGS (depth > 500X), QMPSF	3.50%	11–30% (buccal, swab, neural lesion)
Rofes et al. (2022) [13]	CS	yes	NM_000314.6	c.331 T > C; p.(Trp111Arg)	NGS	22.50%	6–32,5% (sperm, buccal swab, skin)
Hendricks et al. (2022) [20]	CS	yes	NM_000314.6	c.493-2A > G; p?	NGS	< 1% (depth not reported)	21–23% (vascular malformation, buccal swab)
Hendricks et al. (2022) [20]	CS	yes	NM_000314.6	c.284C > T	NGS	11%	12% (buccal swab)
Cavaillé et al. (2023)	CS	yes	NM_000314.6	c.389G > A; p.(Arg130Gln)	NGS	0,7% (17/2285 reads)	12–59% (ganglioneuroma, papilloma, diapneusia fibroid, non-tumoral stomach tissue)

Most of mosaic variants was found from blood samples. Two cases were associated with blood allele frequency < 1%. *CS* Cowden Syndrome, *BRRS* Bannayan-Riley-Ruvalcaba syndrome, *ASD* autism-macrocephaly syndrome, *NGS* Next Generation Sequencing, *MLPA* Multiplex Ligation-dependent Probe Amplification, *aCGH* Array Comparative Genomic Hybridization, *QMPSF*, quantitative multiplex PCR of short fluorescent fragments

As with heterozygous constitutional *PTEN* variants, the entire clinical spectra of *PTEN* can be present as a mosaic, including Cowden syndrome, Bannayan-Riley-Ruvalcaba syndrome, autism-macrocephaly syndrome and Lhermitte–Duclos disease [21]. *PTEN* mosaicism does not seem to be associated with attenuated forms of the pathology: all reported patients except one, and including ours, presented validated clinical criteria. Thus, it is very difficult in practice to distinguish *PTEN* mosaicism from a *priori de novo* PHTS.

The prevalence of detected constitutional *PTEN* variations in patients meeting diagnostic criteria of CS or BRRS is only 30–35% [12]. Moreover, the frequency of de novo variations is significant in this syndromes, estimated at 10.7 to 47.6% of cases [22]. Thus, the absence of identification of variation of interest in patients with CS, in a context of frequent de novo mutations, could be explained using different hypotheses. Firstly, genes other than *PTEN* could be involved. Alternatively, mosaicism for a causal mutation in the *PTEN* gene may not be detectable using routine techniques and approaches.

Concerning the first hypothesis, various studies have identified candidate genes of interest, such as *KLLN*, *AKT1*, *PIK3CA* [23], *SEC23B* [24] or more recently *WWP1* [25]. Although the involvement of these genes was not confirmed at this time by independent laboratories, their exploration may be of interest, with the exception of *AKT1* which remains associated with a particular phenotype (Proteus syndrome), in patients with PTHS criteria not carrying a causal pathogenic variant in *PTEN,* which is not the case in our study.

The second hypothesis depends on the samples analyzed and the analysis techniques. Most of the patients reported with constitutional *PTEN* mosaicism were diagnosed based on the analysis of blood samples. The classical Sanger technique is sensitive enough to find mosaicism with an allelic frequency of about 10%, which was enough to confirm the diagnosis in a number of cases [26].

However, for low-level mosaicisms, NGS and deep sequencing exploration is preferred and it is now performed routinely in many molecular genetics laboratories. The improved sensitivity allowed detection of variants at lower allele frequencies in two PHTS patients of the cohort (1.70% and 3.50% from lymphocytes). In our patient, NGS sequencing failed to detect a variant in DNA from blood, and tumor samples exploration was required. After the identification of the same variant in different tumors and healthy tissue, manual proofreading of the blood sequencing data revealed the causal *PTEN* variant at a frequency of 0.7% (17 / 2285 reads) which is below reasonable sensitivity in diagnostic practice. A similar patient, with a variant undetectable in the blood sample (VAF < 1%) but found in two other tissues (buccal swab, venous malformation), was reported by Hendricks et al. [20].

Deep sequencing (≥ 2000x) in the exploration of mosaicism in hereditary predisposition to cancer is of well-known interest and can detect single nucleotide variants at a frequency of 2–3% in blood samples [26]. However, our study underlines that its use in clinical practice remains difficult in cases with very low mosaicism in blood samples. Indeed, variants with lower allele frequency could only be explored with manual inspection of the aligned reads at hotspots or known positions identified in other samples, as in our patient. Moreover, the detection of variants with very low allele frequency could originate from circulating tumor DNA or a sequencing error. Their interpretation in a diagnostic approach to confirm or not the presence of mosaicism does not therefore seem to be reasonable in routine practice.

Thus, the absence of a pathogenic variant in *PTEN* from blood sample does not rule out the diagnosis of PHTS, and the continued investigation of other tissues must be systematic in patients presenting the testing criteria. This is relevant in this syndrome, because multiple tumoral occurrences are very common. A non-tumor sample could also help to confirm the molecular diagnosis. This is what was done in our study, with the exploration of a non-tumoral stomach tissue sample, confirming the presence of the pathogenic variant *PTEN* outside any context of tumorogenesis, directing on its constitutional character. It should be noted that the cellular composition of buccal swab samples tends to be rich in leukocytes, and should not be considered a different that blood sample [27].

Mosaicism could explain some patients presenting diagnostic criteria for *PTEN* analysis, without identified causal variation. Systematic tumor analysis for these cases, as well as a cohort study, could better estimate the frequency of mosaicism in PHTS. The identification of these patients is important in clinical practice, for their medical management, as well as for that of their family.

Because *PTEN* mosaicism does not seem to be associated with a milder phenotype, the clinical management of concerned patients, including genetic counselling and pre-natal testing, should be the same as that for people with a constitutional heterozygous variant, with the exception of parental testing, which is not useful in the context.

In conclusion, we report a case of *PTEN* mosaicism in a patient diagnosed through DNA sequencing from tumoral and non-tumoral tissue samples. This report highlights the interest of additional tissue analysis when peripheral blood sample DNA sequencing does not identify a causal variant. Based on a review of the literature, we propose that this analysis should now be systematic in this context in clinical practice. Moreover, the literature review provided in this report suggests that *PTEN* mosaicism is not associated with a milder phenotype of the syndrome. The personal and family care of these patients should be identical to that proposed to heterozygous carriers of *PTEN*, with the exception of testing of the parents.

## Data Availability

The datasets used and/or analysed during the current study are available from the corresponding author on reasonable request.

## Abbreviations

ACMG: American College of Medical Genetics
CSF: Cerebrospinal fluid
GATK: Genome Analysis Toolkit
NGS: Next generation sequencing
PHTS: PTEN hamartoma syndrome
*PTEN*: Phosphatase and tensin homolog
SOLAMEN: Segmental outgrowth-lipomatosis-arteriovenous malformation-epidermal nevus
